# Nitrogen Starvation and Stationary Phase Lipophagy Have Distinct Molecular Mechanisms

**DOI:** 10.3390/ijms21239094

**Published:** 2020-11-29

**Authors:** Ravinder Kumar, Muhammad Arifur Rahman, Taras Y. Nazarko

**Affiliations:** 1 Section of Molecular Biology, Division of Biological Sciences, University of California, San Diego, La Jolla, CA 92093, USA; fnu.ravinderkumar@ucsf.edu; 2 Department of Biology, Georgia State University, Atlanta, GA 30303, USA; mrahman27@gsu.edu

**Keywords:** autophagic machinery, autophagy, *Komagataella phaffii*, lipid droplets, lipophagy, *Pichia pastoris*, Prl1, selective autophagy, vacuole, yeast

## Abstract

In yeast, the selective autophagy of intracellular lipid droplets (LDs) or lipophagy can be induced by either nitrogen (N) starvation or carbon limitation (e.g., in the stationary (S) phase). We developed the yeast, *Komagataella phaffii* (formerly *Pichia pastoris*), as a new lipophagy model and compared the N-starvation and S-phase lipophagy in over 30 autophagy-related mutants using the Erg6-GFP processing assay. Surprisingly, two lipophagy pathways had hardly overlapping stringent molecular requirements. While the N-starvation lipophagy strictly depended on the core autophagic machinery (Atg1-Atg9, Atg18, and Vps15), vacuole fusion machinery (Vam7 and Ypt7), and vacuolar proteolysis (proteinases A and B), only Atg6 and proteinases A and B were essential for the S-phase lipophagy. The rest of the proteins were only partially required in the S-phase. Moreover, we isolated the *prl1* (for the positive regulator of lipophagy 1) mutant affected in the S-phase lipophagy, but not N-starvation lipophagy. The *prl1* defect was at a stage of delivery of the LDs from the cytoplasm to the vacuole, further supporting the mechanistically different nature of the two lipophagy pathways. Taken together, our results suggest that N-starvation and S-phase lipophagy have distinct molecular mechanisms.

## 1. Introduction

Autophagy is a highly conserved degradation process in which proteins, protein aggregates, and even entire organelles can be sequestered from the cytoplasm by the vacuoles/lysosomes either directly at the vacuolar/lysosomal membrane (microautophagy) or via the double-membrane vesicular intermediates, autophagosomes (macroautophagy) [1,2]. Autophagy is strongly induced by starvation for nutrients, such as the sources of nitrogen (N), carbon (C), and other elements. The lack of several amino acids can also induce autophagy [3,4]. Therefore, this process acts as an internal supply of building blocks for cells when the external nutrients become unavailable and it allows cells to survive the prolonged periods of starvation.

Lipophagy is an important autophagic process, which delivers the intracellular lipid droplets (LDs) to the vacuoles/lysosomes for degradation and recycling [5]. Lipophagy was initially described in hepatocytes, which become a major site of excessive lipid accumulation in obesity and metabolic syndrome [6]. However, the intracellular lipid metabolism in most eukaryotic cells is also regulated by lipophagy [7], and impaired lipophagy may contribute to the development of many liver and non-liver diseases [8,9]. Thus, understanding the mechanisms of lipophagy is very important for the prevention and treatment of various lipid accumulation diseases.

The budding yeast *Saccharomyces cerevisiae* was used as a simple lipophagy model by several groups to study the mechanisms of lipophagy. Precisely, lipophagy was induced by either acute N-starvation [10,11] or C-limitation (either acute [12] or gradual due to the prolonged incubation of cells in the same medium and entering them into stationary (S) phase [11,13]). These studies described the morphological features of lipophagy and tested the requirements of lipophagy for known autophagy-related (Atg) factors. They suggested that both N-starvation and C-limitation induce microlipophagy [10,11,12,13], the selective microautophagy of LDs, and that this microlipophagy depends on the same core autophagic factors, which are necessary for the formation of autophagic double-membrane in other autophagic pathways [10,12,13]. However, such autophagic membrane was never reported to be associated with LDs in the yeast lipophagy studies questioning the role of the autophagic machinery in yeast lipophagy.

Here, we developed the yeast, *Komagataella phaffii* (formerly *Pichia pastoris*), as a new simple model to study lipophagy. The *K. phaffii* has proven to be an excellent model organism for the studies of autophagy-related (Atg) pathways and contributed a lot of mechanistic insights to the field of autophagy [14]. Then, we run the entire collection of *K. phaffii atg*-mutants through the lipophagy assay under both N-starvation and S-phase conditions. As a result, we found that the core autophagic machinery is essential only for the N-starvation lipophagy. The only overlapping stringent molecular requirements for two lipophagy pathways were Atg6 and vacuolar proteinases A and B. In addition, we isolated a new positive regulator of lipophagy 1 (*prl1*) mutant that was deficient only in the S-phase lipophagy. Therefore, we suggest that the N-starvation and S-phase lipophagy pathways have distinct molecular mechanisms.

## 2. Results

### 2.1. K. phaffii Is a Good Model for Both N-Starvation and S-Phase Lipophagy

**Figure 1:**
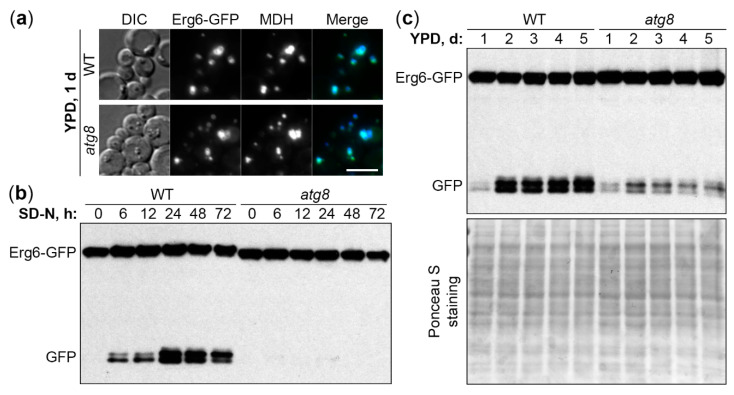
*Komagataella phaffii* is a good model for both N-starvation and S-phase lipophagy. (**a**) *K. phaffii* Erg6-GFP co-localizes with MDH-stained LDs in both WT and *atg8* cells. DIC: Differential interference contrast. Scale bar, 5 µm. (**b**) Atg8 is essential for N-starvation lipophagy. Cells were normalized in SD-N at OD_600_ 1, and an equal volume of culture (1 mL) was processed at all time-points for both strains to nullify the differential growth (Erg6-GFP dilution) effects in SD-N medium (loading control is not applicable). (**c**) Atg8 is only partially required for S-phase lipophagy. Since biomass slightly decreased during the time-course in S-phase, equal biomass (1 OD_600_) was taken at all time-points for both strains. Ponceau S staining was used as a loading control for S-phase samples.

To develop *K. phaffii* as a new lipophagy model, we used the established LD marker protein, Erg6 [10,11,12], tagged with the green fluorescent protein (GFP) on the integrative plasmid, pRK2. To confirm the localization of Erg6-GFP to LDs, wild-type (WT) PPY12h cells with pRK2 integrated into the *HIS4* locus were grown in YPD medium for 1 d to an early S-phase and stained with a blue LD dye, monodansylpentane (MDH) [15]. The Erg6-GFP displayed a complete co-localization with MDH (Figure 1a), suggesting that it is a good LD protein marker for *K. phaffii* under our experimental conditions. The lack of a key Atg protein, Atg8, did not affect the localization of Erg6-GFP to LDs in *atg8* cells (Figure 1a), making it possible to use the Erg6-GFP as a lipophagy reporter.

Then, we developed two Erg6-GFP processing assays to monitor lipophagy: One after the transfer of cells from early S-phase in YPD medium to N-starvation in SD-N medium and another one after the prolonged S-phase in YPD medium. When the LDs with Erg6-GFP are delivered from the cytoplasm to the vacuole, Erg6, but not GFP moiety, is degraded by vacuolar proteases resulting in free GFP, which can be detected by Western blot [16]. The processing of Erg6-GFP to GFP in WT (PPY12h) cells culminated after 24 h of N-starvation (Figure 1b) and after 2-3 days in YPD medium (Figure 1c). Therefore, we picked 0 and 24 h, and 1 and 3 d time-points for the N-starvation and S-phase lipophagy assays, respectively.

Interestingly, while *atg8* cells were completely deficient in the Erg6-GFP processing under N-starvation conditions (Figure 1b), they were only partially compromised in it in S-phase (Figure 1c), suggesting that N-starvation and S-phase lipophagy pathways might have differences in their molecular requirements. In summary, both N-starvation and S-phase lipophagy pathways were readily induced in *K. phaffii* yeast, making it a good model for comparison of their machinery.

### 2.2. Molecular Requirements of N-Starvation and S-Phase Lipophagy in K. phaffii

**Figure 2:**
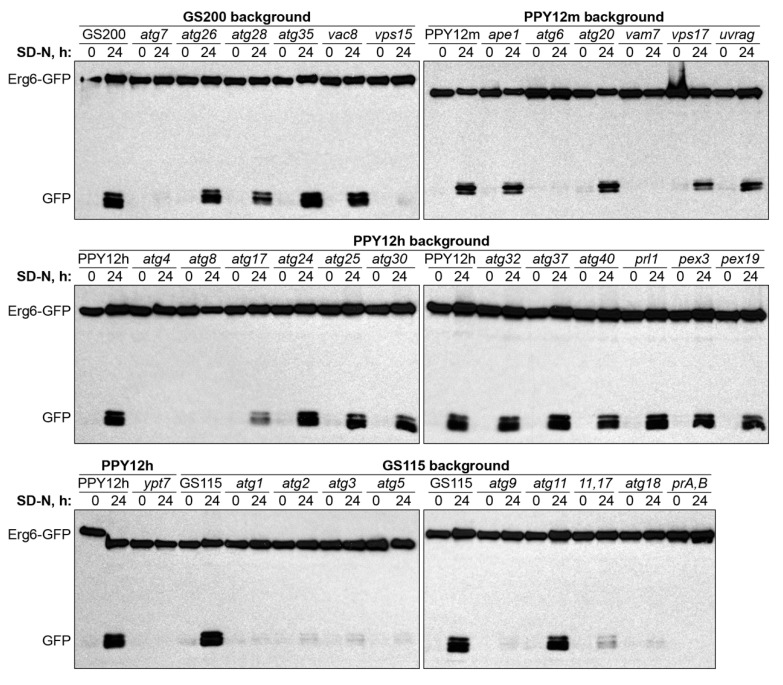
N-starvation lipophagy strictly depends on the core autophagic machinery (Atg1-Atg9, Atg18, and Vps15), vacuole fusion machinery (Vam7 and Ypt7), and vacuolar proteolysis (proteinases A and B). Cells were normalized in SD-N at OD_600_ 1, and an equal volume of culture (1 mL) was processed at both time-points for all strains to nullify the differential growth (Erg6-GFP dilution) effects in SD-N medium (loading control is not applicable). *prA,B:* Proteinases A and B-deficient mutant *pep4 prb1*.

**Figure 3:**
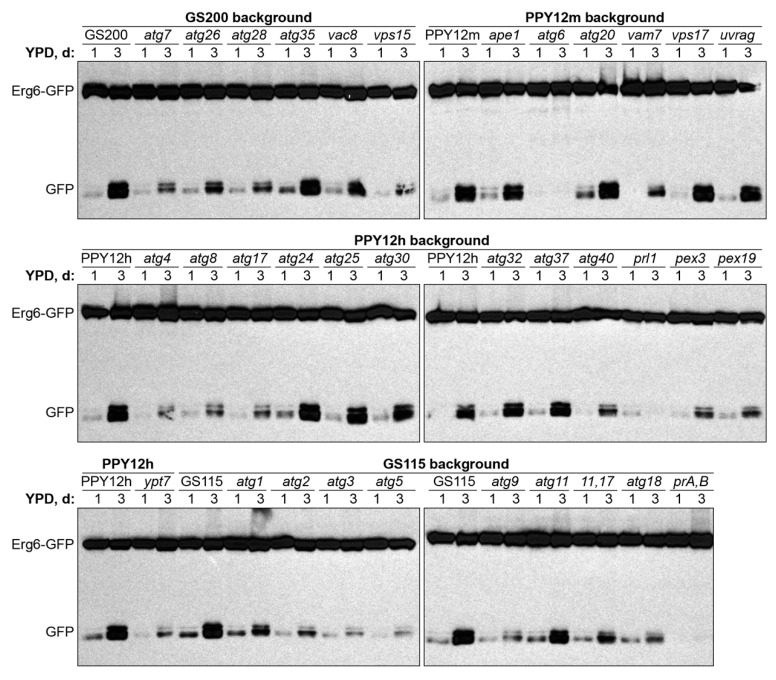
S-phase lipophagy strictly depends only on Atg6, Prl1 (positive regulator of lipophagy 1), and vacuolar proteolysis (proteinases A and B). Since biomass slightly decreased after three days in S-phase, equal biomass (1 OD_600_) was taken at both time-points for all strains. Ponceau S staining was used as a loading control and displayed in Appendix A Figure A1. *prA,B:* Proteinases A and B-deficient mutant *pep4 prb1*.

Encouraged by *atg8* results under two lipophagy conditions, we introduced Erg6-GFP into the collection of *K. phaffii* strains deficient in genes that were previously implicated in various Atg-pathways in either *K. phaffii* or other species (Table 1). The collected mutants belong to 4 different WT backgrounds: GS115, GS200, PPY12h, and PPY12m. Therefore, we grouped mutants by genetic background and studied them together with the corresponding WT strain, as a control, in both N-starvation and S-phase lipophagy conditions (Figure 2 and Figure 3, respectively).

We found that most of the mutants were either fully deficient (*atg1-atg9*, *atg18*, *pep4 prb1*, *vam7*, *vps15*, and *ypt7*) or fully proficient (*ape1*, *atg11*, *atg20*, *atg24-atg26*, *atg30*, *atg32*, *atg35*, *atg37*, *atg40*, *pex3*, *pex19*, *prl1*, *vac8*, *vps17*, and *uvrag*) in the Erg6-GFP processing under N-starvation conditions. Only three strains (*atg17*, *atg11 atg17*, and *atg28*) had an intermediate phenotype (Figure 2 and Table 2). These results suggested that N-starvation lipophagy strictly depends on the core autophagic machinery (Atg1-Atg9, Atg18, and Vps15), vacuole fusion machinery (Vam7 and Ypt7), and vacuolar proteolysis (proteinases A and B).

**Figure A1:**
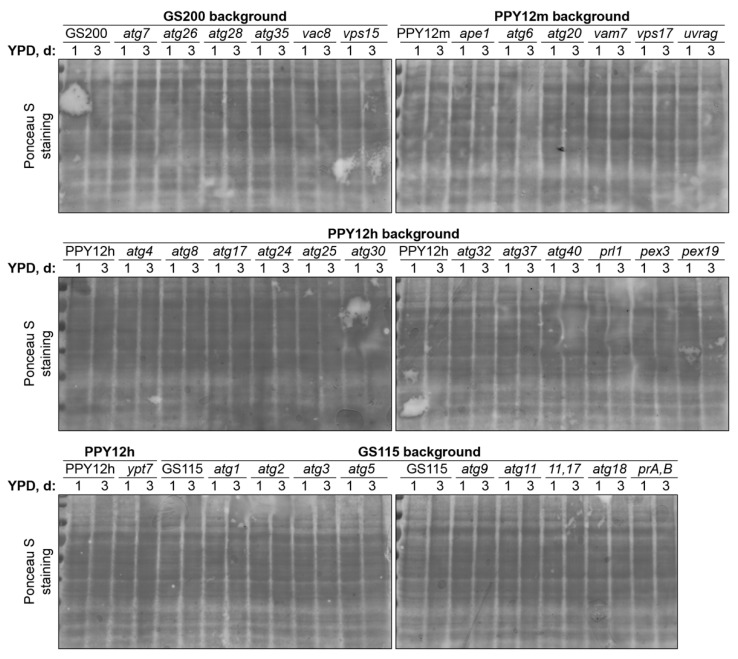
Supplemental figure for Figure 3. Loading control (see Figure 3 for details).

In contrast, the Erg6-GFP processing in S-phase was fully deficient in only three strains (*atg6*, *pep4 prb1*, and *prl1*). It was fully proficient in nearly as many strains (*ape1*, *atg11*, *atg20*, *atg24, atg25*, *atg30*, *atg32*, *atg35*, *atg37*, *vac8*, *vps17*, and *uvrag*), as under N-starvation conditions. However, most of the mutants (*atg1-atg5*, *atg7-atg9*, *atg17*, *atg11 atg17*, *atg18*, *atg26*, *atg28*, *atg40*, *pex3*, *pex19*, *vam7*, *vps15*, and *ypt7*) had an intermediate phenotype (Figure 3 and Appendix A Figure A1; Table 2). Therefore, we concluded that S-phase lipophagy strictly depends only on Atg6, Prl1, and vacuolar proteolysis (proteinases A and B). Summarizing, the N-starvation and S-phase lipophagy pathways have different molecular requirements.

### 2.3. Prl1 Is Essential for the Delivery of LDs to the Vacuole in S-Phase

To probe further into the differences between N-starvation and S-phase lipophagy machinery, we took advantage of the *prl1* mutant. This mutant was obtained by integrating *Zeocin^R^* cassette from the pRK6 plasmid into the genome of PPY12h WT strain (Table 1). The *prl1* mutant displayed a unique phenotype in the screening above: It was fully proficient in the N-starvation lipophagy, but fully deficient in the S-phase lipophagy (Table 2).

**Figure 4:**
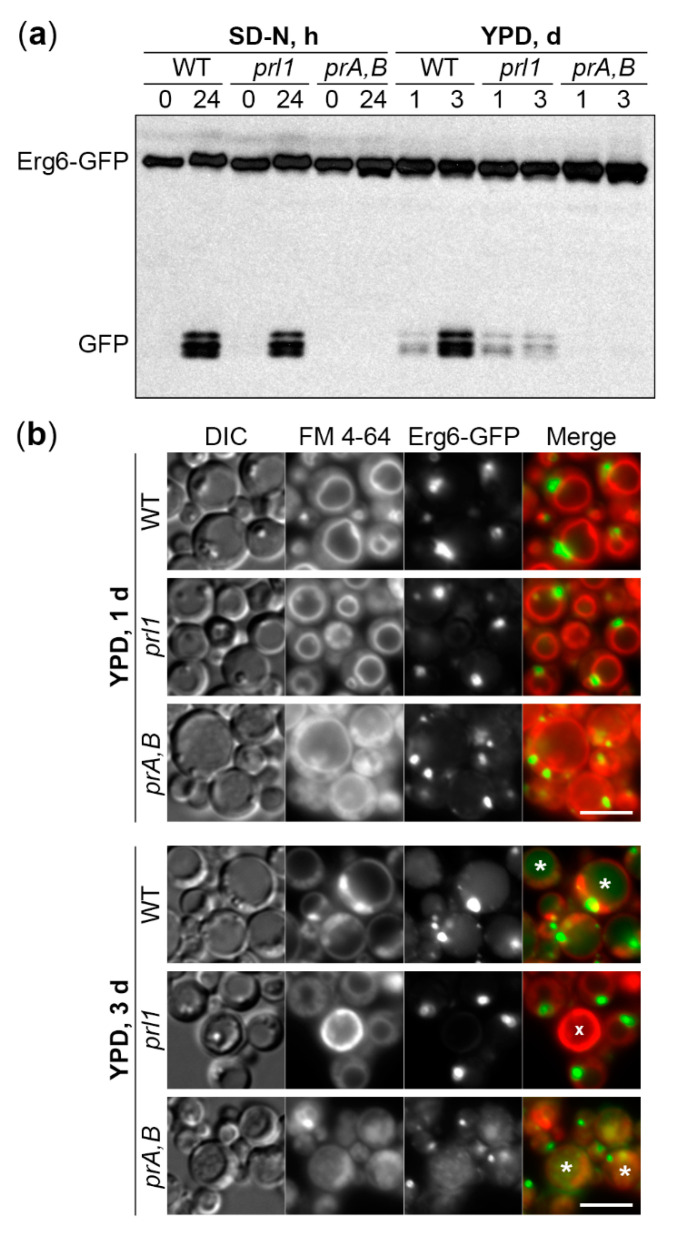
Prl1 is essential to deliver LDs to the vacuole in S-phase. (**a**) S-phase, but not N-starvation lipophagy depends on Prl1. N-starvation and S-phase cells were processed as described in Figure 2 and Figure 3, respectively. Ponceau S staining (Appendix A Figure A2) was used as a loading control for S-phase samples. (**b**) Prl1 is required to deliver LDs to the vacuole in S-phase. Vacuole membranes were stained red with FM 4-64. DIC: Differential interference contrast; *prA,B:* Proteinases A and B-deficient mutant *pep4 prb1*; *: GFP-containing vacuole; x: Dead cell. Scale bar, 5 µm.

**Figure A2:**
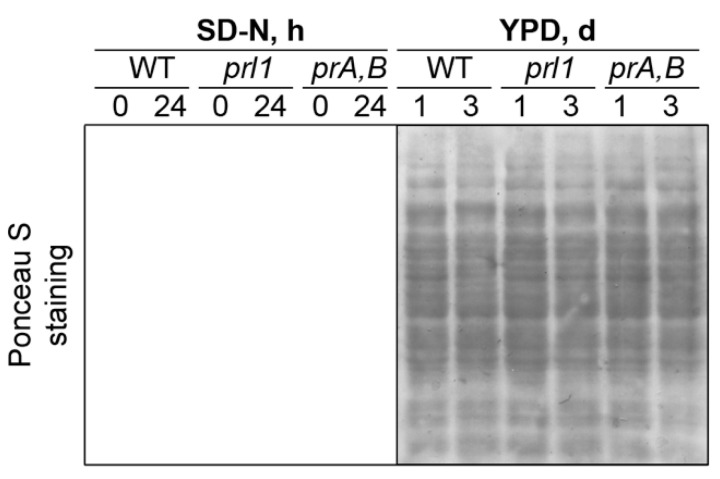
Supplemental figure for Figure 4a. Loading control (see Figure 4a for details).

To compare the phenotypes of *prl1* cells in the same experiment, we split the cultures of WT, *prl1*, and *pep4 prb1* cells after 1 d in YPD medium: Small aliquots were transferred to SD-N medium (for 0 and 24 h time-points), while the rest remained in YPD medium (for 1 and 3 d time-points) (Figure 4a and Appendix A Figure A2). While N-starvation and S-phase lipophagy pathways were equally well induced in WT cells, they were fully blocked in the proteinases A and B-deficient mutant. Although *prl1* cells were indistinguishable from WT cells under N-starvation conditions, they were indeed incapable of degrading LDs in S-phase (Figure 4a). Therefore, we concluded that the *prl1* mutant is specifically deficient in the S-phase lipophagy.

To get insight into the step of S-phase lipophagy affected in *prl1* cells, we studied S-phase lipophagy by fluorescence microscopy. The WT, *prl1*, and *pep4 prb1* cells with Erg6-GFP reporter were incubated in the YPD medium with FM 4-64, the lipophilic dye that stains specifically the vacuolar membrane. After 1 d, the cells of all strains had LDs outside the vacuole and no GFP fluorescence inside the vacuolar lumen. However, after 3 d, WT and *pep4 prb1* cells developed a diffuse or grainy GFP fluorescence in the vacuolar lumen, respectively (Figure 4b). Those patterns of luminal GFP fluorescence were consistent with the disintegration of the LD-containing autophagic bodies in WT vacuoles and Brownian movement of intact LD-containing autophagic bodies in the proteinases A and B-deficient vacuoles. The *prl1* cells did not gain any GFP fluorescence in the vacuolar lumen after 3 d in YPD, suggesting that Prl1 is required to deliver LDs to the vacuole in S-phase.

## 3. Discussion

In this study, we introduced *K. phaffii* yeast as a new lipophagy model and compared the lipophagy requirements of *K. phaffii* cells under two conditions: N-starvation and S-phase, the two most popular ways to induce Atg-pathways in yeast. Previous screenings in *S. cerevisiae* done for each of these conditions separately indicated that both of them induced microlipophagy that strongly depended on the core autophagic machinery [10,13]. However, by comparing the N-starvation and S-phase conditions in the same study with *K. phaffii*, we observed a clear difference in lipophagy requirements (Table 2).

Both the N-starvation and S-phase lipophagy pathways strictly depended on the proteinases A and B, consistent with the vacuolar degradation of LDs under the two conditions. While the N-starvation lipophagy strongly relied on the core autophagic machinery represented by Atg1-Atg9, Atg18, and Vps15, the S-phase lipophagy was fully deficient only without the Atg6 protein. Interestingly, only Atg6 and not the other components of the phosphatidylinositol 3-kinase complex I (i.e., Atg14, Atg38, Vps15, and Vps34) stably localized to the vacuolar membrane under both acute and gradual (S-phase) C-limitation conditions in *S. cerevisiae* [12]. Atg6 was necessary for the formation of raft-like domains in the vacuolar membrane [12] that are essential for microlipophagy [13]. Combined, this and previous studies suggest that Atg6 plays a unique role in the S-phase lipophagy, which is different from its established function in the biogenesis of autophagic double-membrane under N-starvation conditions [39].

While a reason for the essential role of the core autophagic machinery in the N-starvation lipophagy is unclear, there is a plausible explanation for the partial requirement of the core autophagic machinery in the S-phase lipophagy. In S-phase, the core autophagic machinery is essential for the correct vacuolar localization of Niemann-Pick type C proteins, Ncr1 and Npc2, which transport sterol from the vacuolar lumen to the vacuolar membrane for the raft-like domain formation [11]. However, the requirements of Ncr1 and Npc2 for (1) raft-like domains formation, (2) their internalization as microautophagic bodies, and (3) S-phase lipophagy are partial [11]. Therefore, the core autophagic machinery has, in the end, a partial role in the S-phase lipophagy. Interestingly, it is not required for the correct vacuolar localization of Ncr1 and Npc2 under the N-starvation conditions. Thus, the mechanistic role of the core autophagic machinery in the N-starvation lipophagy is still unknown.

Recently, it was reported that the vacuolar membrane protein, Vph1, which is normally excluded from the raft-like domains in S-phase [40], is also degraded, like LDs, by microautophagy [41]. Interestingly, the S-phase microautophagy of Vph1 was independent of the core autophagic machinery, but relied on the machinery of ESCRT, the endosomal sorting complex required for transport. The same study also reported that the S-phase microautophagy of LDs was partially independent of the core autophagic factor, Atg1, but strongly relied on the ESCRT component, Vps27 [41]. Our results are consistent with these lipophagy observations and extend them to the entire core autophagic machinery being only partially required specifically in the S-phase. However, it is still unclear how LDs and Vph1 can utilize the same microautophagy pathway in S-phase, since they are associated with different vacuolar membrane domains, the raft-like liquid-ordered domain and the liquid-disordered domain, respectively. Since the S-phase microautophagy of Vph1 does not require Atg6 [41], and the S-phase lipophagy strongly depends on it (Figure 3), we propose that these pathways have both common (Vps27) and unique (Atg6) requirements.

Our study also suggests a unique molecular requirement of the S-phase lipophagy versus N-starvation lipophagy, the positive regulator of lipophagy 1 (Prl1). The *prl1* mutant isolated in this study was deficient in lipophagy only in the S-phase. Moreover, we showed that the *prl1′s* lipophagy block is at a trafficking step, since LDs were not delivered from the cytoplasm to the vacuole for degradation in the S-phase. It will be interesting to determine the gene responsible for *prl1* phenotype, since it can help us to further distinguish the molecular mechanisms of these two clearly distinct lipophagy pathways, the N-starvation and S-phase lipophagy.

## 4. Materials and Methods

### 4.1. Strains and Plasmids

The *K. phaffii* strains that were used in this study are shown in Table 1. These strains were transformed by electroporation [42] with the EcoNI-linearized (R0521S; New England Biolabs, Ipswich, MA, USA) pRK2 plasmid. This plasmid contained the Erg6-GFP expression cassette (for lipophagy studies) and *HIS4* marker gene (for integration into *his4* mutant allele of the recipient strains and selection of His^+^-transformants). The resulting transformants had the following genotype: *his4*::pRK2 (P*_ERG6_-ERG6-GFP*, *HIS4*). They were selected on SD+CSM-His plates (1.7 g/L YNB without amino acids and ammonium sulfate, 20 g/L dextrose, 5 g/L ammonium sulfate, 0.78 g/L CSM-His, and 20 g/L agar) and screened for expression of Erg6-GFP by Western blot with anti-GFP bodies (11814460001; Roche Diagnostics, Mannheim, Germany) and for localization of Erg6-GFP to LDs by fluorescence microscopy.

### 4.2. Fluorescence Microscopy

Cells were grown for 1 and/or 3 d in culture tubes with 1 mL of YPD medium (10 g/L yeast extract, 20 g/L peptone and 20 g/L dextrose; the autoclaved solution of yeast extract and peptone was mixed with the filter-sterilized 20× solution of dextrose). LDs were stained with 1 µL of 0.1 M MDH solution (SM1000a; Abcepta, San Diego, CA, USA) during the last 1 h of incubation of cells in YPD medium. Vacuolar membranes were stained with 1 µL of 1 mg/mL solution of FM 4-64 (T3166; Molecular Probes, Eugene, OR, USA) in DMSO added at the beginning of incubation of cells in YPD medium. Then, cells were immobilized on slides using 1% low-melt agarose. For this, the 2 µL drop of cell culture on the slide was mixed with the 5 µL drop of 1% low-melt agarose (37 °C) on the coverslip. Microscopy was done at the Axioskop 2 MOT microscope equipped with the Plan-Apochromat 100×/1.40 NA oil DIC objective and operated by the AxioVision software (Carl Zeiss Microscopy, White Plains, NY, USA). All the experiments were done at least in duplicate.

### 4.3. Biochemical Studies

Cells were grown in culture tubes with 1 mL of YPD medium and 1 OD_600_ of cells was taken at 1 and 3 d time-points for studies of S-phase lipophagy. For studies of N-starvation lipophagy, 3 OD_600_ of cells were taken at 1 d time-point in YPD medium, washed twice with 1 mL of 1× YNB solution (1.7 g/L YNB without amino acids and ammonium sulfate), and resuspended in 3 mL of SD-N medium (1.7 g/L YNB without amino acids and ammonium sulfate, and 20 g/L dextrose). Then, 1 mL of cell culture was taken at 0 and 24 h time-points in SD-N medium. Both YPD (1 and 3 d) and SD-N (0 and 24 h) samples were TCA precipitated [43] and analyzed by Western blot with the same anti-GFP bodies, as above. All the experiments were done at least in duplicate.

## Figures and Tables

**Table 1:** *K. phaffii* strains used in this study.

Mutant Name	Strain Name	Background	Genotype and Plasmid	Source
WT	GS115	GS115	*his4*	[17]
WT	GS200	GS200	*arg4 his4*	[18]
WT	PPY12h	PPY12h	*arg4 his4*	[19]
WT	PPY12m	PPY12m	*arg4 his4*	[19]
*ape1*	SJCF434	PPY12m	∆*ape1*::*Geneticin^R^ arg4 his4*	[20]
*atg1*	R12	GS115	*atg1-1*::*Zeocin^R^ his4*	[21]
*atg2*	WDK011	GS115	∆*atg2*::*Zeocin^R^ his4*	[21]
*atg3*	*gsa20*	GS115	*atg3*::*Zeocin^R^ his4*	[21]
*atg4*	PPM408	PPY12h	*atg4*::*Zeocin^R^ arg4 his4*	[22]
*atg5*	SJCF2320	GS115	∆*atg5*::*Zeocin^R^ his4*	SL ^1^
*atg6*	SRDM006	PPY12m	∆*atg6*::*Geneticin^R^ arg4 his4*	[23]
*atg7*	WDK07	GS200	∆*atg7*::*ScARG4 arg4 his4*	[24]
*atg8*	SJCF925	PPY12h	∆*atg8*::*Geneticin^R^ arg4 his4*	[25]
*atg9*	R19	GS115	*atg9-1*::*Zeocin^R^ his4*	[21]
*atg11*	R8	GS115	*atg11-2*::*Zeocin^R^ his4*	[26]
*atg17*	SJCF929	PPY12h	∆*atg17*::*Geneticin^R^ arg4 his4*	[25]
*atg11 atg17*	SJCF948	GS115	*atg11-2*::*Zeocin^R^* ∆*atg17::Geneticin^R^ his4*	[25]
*atg18*	R2	GS115	*atg18-1*::*Zeocin^R^ his4*	[21]
*atg20*	SRDM020	PPY12m	∆*atg20*::*Geneticin^R^ arg4 his4*	SL
*atg24*	*paz16*	PPY12h	*atg24*::*Zeocin^R^ arg4 his4*	[27]
*atg25*	SJCF1231	PPY12h	∆*atg25*::*Geneticin^R^ arg4 his4*	[23]
*atg26*	∆*pdg3*	GS200	∆*atg26*::*ScARG4 arg4 his4*	[28]
*atg28*	∆*pdg2*	GS200	∆*atg28*::*ScARG4 arg4 his4*	[29]
*atg30*	SJCF936	PPY12h	∆*atg30*::*Zeocin^R^ arg4 his4*	[25]
*atg32*	SJCF1715	PPY12h	∆*atg32*::*Geneticin^R^ arg4 his4*	[30]
*atg35*	SVN1	GS200	∆*atg35*::*ScARG4 arg4 his4*	[31]
*atg37*	STN96	PPY12h	∆*atg37*::*Geneticin^R^ arg4 his4*	[32]
*atg40*	SRK2	PPY12h	∆*atg40*::*Zeocin^R^* (pRK4) *arg4 his4*	This study
*pep4 prb1*	SMD1163	GS115	*pep4 prb1 his4*	[33]
*pex3*	SEW1	PPY12h	∆*pex3*::*PpARG4 arg4 his4*	[34]
*pex19*	SKF13	PPY12h	∆*pex19*::*Zeocin^R^ arg4 his4*	[35]
*prl1*	SRK3	PPY12h	*prl1*::*Zeocin^R^* (pRK6) *arg4 his4*	This study
*vac8*	WDY53	GS200	∆*vac8*::*Zeocin^R^ arg4 his4*	[36]
*vam7*	SRDM050	PPY12m	∆*vam7*::*Zeocin^R^ arg4 his4*	[37]
*vps15*	OP5	GS200	∆*vps15*::*ScARG4 arg4 his4*	[38]
*vps17*	SRDM122	PPY12m	∆*vps17*::*Geneticin^R^ arg4 his4*	[23]
*uvrag*	SRDM083	PPY12m	∆*uvrag*::*Zeocin^R^ arg4 his4*	[23]
*ypt7*	SRRM197	PPY12h	∆*ypt7*::*Geneticin^R^ arg4 his4*	[37]

^1^ SL: Subramani laboratory.

**Table 2:** Lipophagy phenotype of *K. phaffii* (this study) and *S. cerevisiae* mutants.

Strain (*Kp*/*Sc*)	*Kp* (This Study)		*Sc* [10]		*Sc* [13]	*Sc* [12]
	SD-N	S-Phase	SD-N	SD-N	S-Phase	SD-D (0.4%)
	Erg6-GFP	Erg6-GFP	Erg6-GFP	Faa4-GFP	BODIPY	Erg6-DsRed
WT	+	+	+	+	+	+
*ape1*	+	+	ND ^1^	ND	ND	ND
*atg1*	−	+/−	−	−	−	−
*atg2*	−	+/−	ND	ND	−	−
*atg3*	−	+/−	−	−	−	−
*atg4*	−	+/−	−	−	−	ND
*atg5*	−	+/−	−	−	−	−
*atg6*	−	−	−	−	−	−
*atg7*	−	+/−	−	−	−	−
*atg8*	−	+/−	−	−	−	−
*atg9*	−	+/−	−	−	−	−
*atg11*	+	+	ND	+/−	+	+
*atg17*	+/−	+/−	ND	−	−	+/−
*atg11 atg17*	+/−	+/−	ND	ND	ND	ND
*atg18*	−	+/−	−	−	−	−
*atg20*	+	+	ND	+	+	+
*atg24*	+	+	ND	ND	+	+
*atg25*	+	+	NA ^2^	NA	NA	NA
*atg26*	+	+/−	ND	ND	+	+
*atg28/atg29,31*	+/−	+/−	ND	ND	−, −	+/−, +/−
*atg30/atg36*	+	+	ND	ND	+	+
*atg32*	+	+	ND	ND	−	+/−
*atg35*	+	+	NA	NA	NA	NA
*atg37*	+	+	NA	NA	NA	NA
*atg40*	+	+/−	ND	ND	ND	ND
*pep4 prb1*	−	−	ND	ND	−	ND
*pex3*	+	+/−	ND	ND	ND	ND
*pex19*	+	+/−	ND	ND	ND	ND
*prl1*	+	−	NA	NA	NA	NA
*vac8*	+	+	ND	−	ND	ND
*vam7*	−	+/−	−	−	ND	ND
*vps15*	−	+/−	ND	ND	ND	ND
*vps17*	+	+	ND	ND	ND	ND
*uvrag/vps38*	+	+	+/−	+/−	ND	ND
*ypt7*	−	+/−	−	−	ND	ND

^1^ ND: Phenotype not determined in *Sc*; ^2^ NA: Mutant not available in *Sc*. “ + “: Fully proficient in lipophagy; “ – “: Fully deficient in lipophagy; “ +/− “: Intermediate phenotype.

## Abbreviations

Atg	autophagy-related
C	carbon
DIC	differential interference contrast
ESCRT	endosomal sorting complex required for transport
GFP	green fluorescent protein
LD	lipid droplet
MDH	monodansylpentane
N	nitrogen
Prl1	positive regulator of lipophagy 1
prA,B	proteinases A and B
S	stationary
WT	wild-type
